# High-resolution crystal structure of the Mu8.1 conotoxin from *Conus mucronatus*

**DOI:** 10.1107/S2053230X23007070

**Published:** 2023-08-29

**Authors:** Emilie Müller, Celeste Menuet Hackney, Lars Ellgaard, Jens Preben Morth

**Affiliations:** a Department of Biotechnology and Biomedicine, Technical University of Denmark, 2800 Kongens Lyngby, Denmark; b Department of Biology, University of Copenhagen, 2200 Copenhagen, Denmark; European Molecular Biology Laboratory, France

**Keywords:** conotoxins, zinc binding, toxins, hydrogen bonding, Mu8.1

## Abstract

Marine cone snails produce a wealth of peptide toxins (conotoxins) that bind their molecular targets with high selectivity and potency. Therefore, conotoxins constitute valuable biomolecular tools with a variety of biomedical purposes. The Mu8.1 conotoxin from *Conus mucronatus* is the founding member of the newly identified saposin-like conotoxin class of conotoxins and has been shown to target Cav2.3, a voltage-gated calcium channel. Two crystal structures have recently been determined of Mu8.1 at 2.3 and 2.1 Å resolution. Here, a high-resolution crystal structure of Mu8.1 was determined at 1.67 Å resolution in the high-symmetry space group *I*4_1_22. The asymmetric unit contained one molecule, with a symmetry-related molecule generating a dimer equivalent to that observed in the two previously determined structures. The high resolution allows a detailed atomic analysis of a water-filled cavity buried at the dimer interface, revealing a tightly coordinated network of waters that shield a lysine residue (Lys55) with a predicted unusually low side-chain p*K*_a_ value. These findings are discussed in terms of a potential functional role of Lys55 in target interaction.

## Abstract 2

The crystal structure of the Mu8.1 conotoxin was determined in the high-symmetry space group *I*4_1_22 at 1.67 Å resolution. This crystal structure reveals a surface-exposed Zn^2+^-binding site and establishes a hydrogen-bonding network around Lys55 buried at the dimer interface.

## 1. Introduction

Animal venom peptides and proteins constitute a rich source of bioactive compounds that are used as research tools and provide new drug candidates. Several venom peptides have already been commercialized and used to treat a range of conditions, such as hypertension, chronic pain and thrombosis (Smallwood & Clark, 2021 ▸). Predatory marine cone snails produce a wide variety of venom peptides, known as conopeptides or conotoxins, with as many as 200 000 different peptides estimated to exist in nature (Lin *et al.*, 2021 ▸). Conotoxins are produced in the endoplasmic reticulum of cells in the cone-snail venom gland and most have a mature sequence ranging between 15 and 30 residues (Safavi-Hemami *et al.*, 2018 ▸). Thus, like other peptide toxins, conotoxins are typically stabilized by disulfide bonds (Undheim *et al.*, 2016 ▸). While many of these small peptides are well characterized and known to target various cell-surface proteins, such as ion channels, different receptors and transporters, larger conotoxins (also known collectively as macro-conotoxins), such as Mu8.1 (89 residues), generally remain understudied.

The great potential of conotoxins as bioactive compounds is underscored by the recent identification in cone-snail venom of nature’s shortest insulin molecules (43 residues for Con-Ins G1 from *Conus geographus* compared with 51 residues for human insulin) that show high structural similarity to human insulin (Safavi-Hemami *et al.*, 2015 ▸; Menting *et al.*, 2016 ▸). These weaponized insulins can activate the human insulin receptor and lower blood glucose in zebrafish and mouse models of diabetes, indicating a potential future use as fast-acting drugs in the treatment of diabetes (Ahorukomeye *et al.*, 2019 ▸). Another interesting class of conotoxins are the ‘con-ikot-ikot’ toxins that inhibit the desensitization of the GluA2 AMPA receptor (AMPAR; Walker *et al.*, 2009 ▸). This inter­action has been explored by structural analysis of the crystal structure of the complex between GluA2 and con-ikot-ikot from *C. striatus*, in turn providing new insights into receptor function (Chen *et al.*, 2014 ▸; Baranovic *et al.*, 2022 ▸).

Two recently published crystal structures of *C. mucronatus* Mu8.1 revealed an all-helical molecule forming a homodimer, with a common dimer interface observed in two different crystal conditions (Hackney *et al.*, 2023 ▸). Dimer formation was supported by small-angle X-ray scattering (SAXS) and gel-filtration data showing that the majority of Mu8.1 also formed a stable dimer in solution. These observations suggested that dimeric Mu8.1 is likely to be of biological relevance. Each protomer in the dimer comprises five disulfide bonds connecting the four α-helices and shows structural similarity to saposin-like proteins as well as con-ikot-ikot from *C. striatus* (Hackney *et al.*, 2023 ▸).

Here, we present a high-resolution crystal structure of Mu8.1 obtained from a new crystal condition in the high-symmetry space group *I*4_2_22 at 1.67 Å resolution. The structure reveals a dimer interface equivalent to that previously observed in Mu8.1; however, in the present crystal structure it is formed between two symmetry-related molecules. We further explore the water-coordinated pocket shielded by the dimer interface. The network of water molecules revealed at 1.67 Å resolution surrounds a potential functional residue, Lys55, and may contribute to the unusually low p*K*_a_ value (8.16) predicted for Lys55 by *PROPKA* (Søndergaard *et al.*, 2011 ▸; Olsson *et al.*, 2011 ▸). Moreover, a surface-exposed divalent cation-binding site with a bound Zn^2+^ ion that mediates crystal contacts between two dimers was identified on the ‘backside’ of Mu8.1, opposite the dimer interface.

## 2. Materials and methods

### 2.1. Expression and purification of Mu8.1

Expression and purification of Mu8.1 followed the protocol described in Hackney *et al.* (2023 ▸). Briefly, Ub-His_10_-Mu8.1 was expressed from a pET-39_Ub19 vector co-transformed into *Escherichia coli* BL21 cells with the csCyDisCo plasmid (pLE577; Nielsen *et al.*, 2019 ▸). Following overnight expression in auto-induction medium at 25°C, the resulting cell pellets were resuspended in 5 ml lysis buffer (50 m*M* Tris pH 8, 300 m*M* NaCl, 20 m*M* imidazole) per gram of cell pellet. Resuspended cells were lysed by sonication while keeping the lysate on ice throughout. Ub-His_10_-Mu8.1 was purified from the soluble cellular fraction by Ni–NTA immobilized metal-affinity chromatography (IMAC) using a linear elution gradient from 0% to 100% in IMAC buffer (50 m*M* Tris pH 8, 300 m*M* NaCl, 400 m*M* imidazole). Peak fractions were dialyzed twice against 2 l anion-exchange (AEX) buffer (50 m*M* NaH_2_PO_4_/Na_2_HPO_4_ pH 6.8, 20 m*M* NaCl) and the sample was subjected to AEX chromatography on a 10/100 Tricorn column (Cytiva) packed with Source 15Q ion-exchange resin (Amersham Biosciences, GE Healthcare). The bound protein sample was washed with 15% AEX elution buffer (50 m*M* NaH_2_PO_4_/Na_2_HPO_4_ pH 6.8, 1 *M* NaCl) until a stable UV baseline was observed. Ub-His_10_-Mu8.1 was then eluted over six column volumes using a gradient from 15% to 50% AEX elution buffer. The eluted fusion protein was incubated with preactivated His-tagged Tobacco etch virus (TEV) protease in a 1:20 ratio overnight at room temperature. The TEV protease-cleaved Ub-His_10_-Mu8.1 was subsequently loaded onto 8 ml Talon cobalt resin (Takara) pre-equilibrated in AEX buffer. The flowthrough containing untagged Mu8.1 was pooled and finally subjected to size-exclusion chromatography on a Superdex 75 Increase 10/300 GL column (Cytiva) pre-equilibrated in 200 m*M* NH_4_HCO_3_ buffer pH 7.8. Fractions containing pure Mu8.1 were pooled and lyophilized. The purity was estimated by SDS–PAGE to be >95% (Hackney *et al.*, 2023 ▸). The final yield was estimated to be 2 mg per litre of cell culture.

### 2.2. Crystallization

Freeze-dried Mu8.1 was dissolved in Milli-Q ultrapure water to a concentration of 5 mg ml^−1^. The concentration was measured with a NanoDrop 1000 using a predicted extinction coefficient of 12 010 *M* ^−1^ cm^−1^. Crystallization was performed by the hanging-drop vapor-diffusion method at 21°C. Drops were set up at a 1:1 ratio of reservoir solution to protein solution in a total volume of 2 µl in 24-well drop format on siliconized glass cover slides. The wells were sealed with immersion oil (Sigma–Aldrich, catalogue No. 56822) and equilibrated against 500 µl reservoir solution at 21°C. Crystals of Mu8.1 appeared in several conditions from the LMB Crystallization Screen (Molecular Dimensions, catalogue No. MD1-98; Gorrec, 2009 ▸) in a few days to weeks.

The best diffracting crystals were obtained from box 2 condition 21 of the LMB Crystallization Screen consisting of 18%(*w*/*v*) polyethylene glycol (PEG) 5000 monoethyl ether (MME), 0.2 *M* ammonium sulfate, 0.1 *M* 2-(*N*-morpholino)ethanesulfonic acid (MES) pH 6.5 adjusted with NaOH. Crystals were harvested using mounted CryoLoops (Hampton Research) with cryoprotection performed by quickly dipping the crystal into ∼17% ethylene glycol (Teng & Moffat, 1998 ▸) prepared by mixing 1 µl 50% ethylene glycol with 2 µl reservoir solution. The crystals were flash-cooled in liquid nitrogen and shipped to the beamline for remote data collection. Crystallization information is summarized in Table 1 ▸.

### 2.3. Data collection and processing

Diffraction data collection was carried out on the BioMax beamline at MAX IV, Lund, Sweden (Ursby *et al.*, 2020 ▸). Data were collected at 100 K for a full sweep of 360° with an oscillation of 0.1°, with 0.011 s exposure time, at 12 700 eV. To reduce potential radiation damage, only part of the data set was processed (140°) sufficient for a highly complete data set. Data-collection and processing statistics are summarized in Table 2 ▸.

### 2.4. Structure solution and refinement

The structure of Mu8.1 was determined by molecular replacement with *Phaser-MR* (McCoy *et al.*, 2007 ▸) using the recently published lower resolution crystal structure of Mu8.1 as a search model (PDB entry 7px2; Hackney *et al.*, 2023 ▸).

Model building and refinement were performed with *phenix.refine* (Adams *et al.*, 2010 ▸) with iterative rebuilding in *Coot* (Emsley *et al.*, 2010 ▸). Refinement statistics are summarized in Table 3 ▸. Coordinates/structure factors have been submitted to the PDB with accession code 8amy.

Molecular graphics were presented with the *PyMOL* molecular-graphics system (version 2.2r7pre; Schrödinger). Prediction of p*K*_a_ values was performed by *PROPKA* version 3.0 (https://www.ddl.unimi.it/vegaol/propka.htm; Olsson *et al.*, 2011 ▸; Søndergaard *et al.*, 2011 ▸). The metal ion was identified using the metal-binding site validation server *CheckMyMetal* (https://cmm.minorlab.org/; Zheng *et al.*, 2014 ▸). Mu8.1 was refined with both a Zn^2+^ ion and an Ni^2+^ ion in the surface metal site. As input, a PDB file including both the Mu8.1 coordinates and a symmetry-equivalent molecule completing the metal site were uploaded. The coordinate *B* factor of the metal ion, the liganding residues in the nearby environment and the tetragonal geometry were compared and all favored a Zn^2+^ ion (Kuppuraj *et al.*, 2009 ▸).

## 3. Results and discussion

### 3.1. A disulfide network contributes to the rigidity of the Mu8.1 protomer

Here, we crystallized Mu8.1 in a new crystal condition and determined the structure at 1.67 Å resolution, the highest resolution reported to date. The structure was refined to a final *R*_work_ and *R*_free_ of 0.169 and 0.200, respectively (Table 3 ▸). *MolProbity* reports no Ramachandran outliers, with 96.4% of the residues in favored regions (Table 3 ▸).

**Figure 1:**
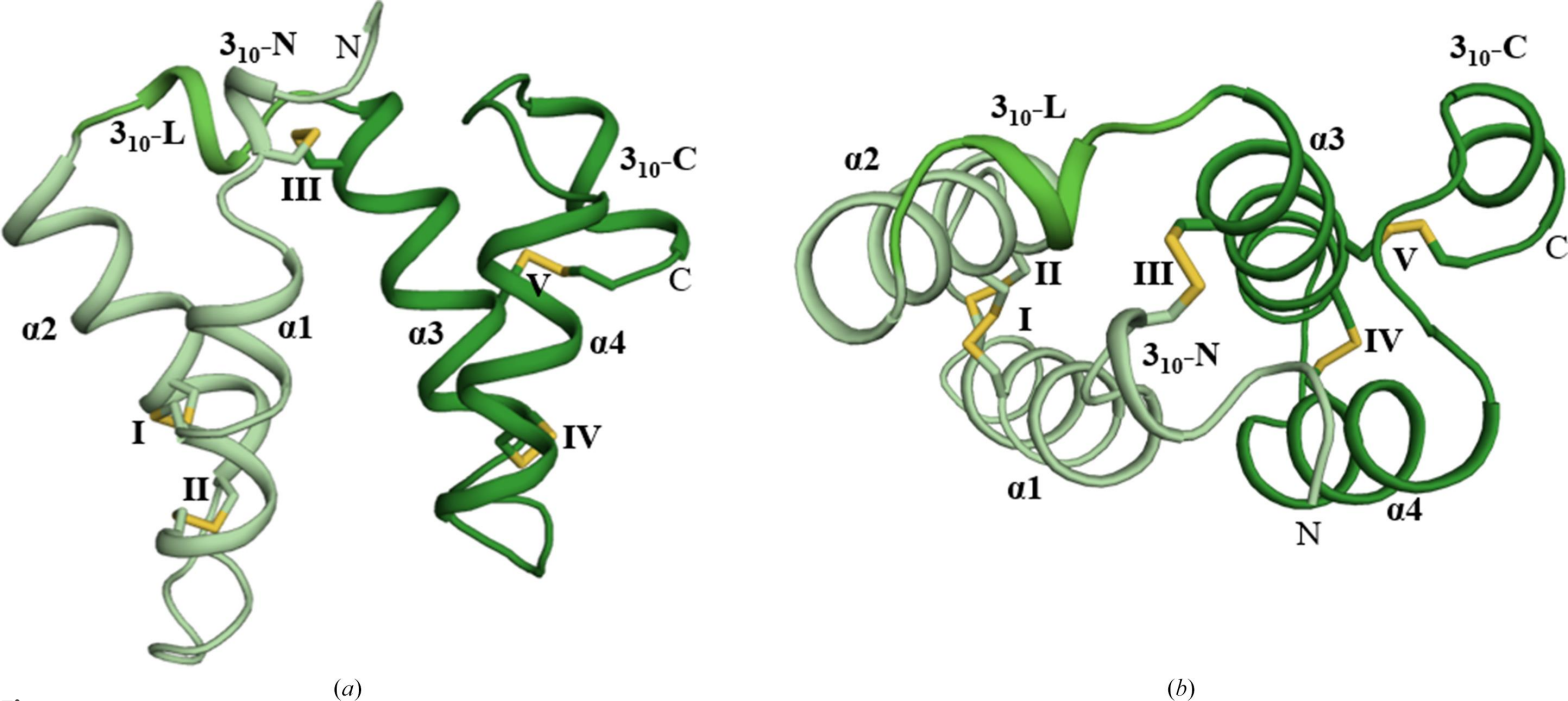
High-resolution crystal structure of Mu8.1. (*a*) Cartoon presentation of the Mu8.1 promoter observed in the asymmetric unit in space group *I*4_1_22. α-Helices (α1–α4) are numbered consecutively from the N-terminus to the C-terminus (labeled ‘N’ and ‘C’). 3_10_-Helices are labeled according to their N-terminal (3_10_-N), linker (3_10_-L) or C-terminal (3_10_-C) position. Disulfides are presented as yellow sticks and labeled with roman numerals. The first domain is colored pale green, 3_10_-L is colored light green and the second domain is colored dark green. (*b*) Top view of Mu8.1 with the same labeling, numbering and coloring as in (*a*).

The asymmetric unit accommodates a single protomer comprising two helical ‘domains’ that interact to form a hydrophobic core (Fig. 1 ▸ *a*). The first domain comprises an N-terminal 3_10_-helix (3_10_-N) followed by α-helix 1 (α1) and α2 (Figs. 1 ▸ *a* and 1 ▸ *b*). The second domain comprises α3–α4 followed by a C-terminal 3_10_-helix (3_10_-C). The two domains are linked by a 3_10_-helix linker (3_10_-L). Five disulfide bonds contribute to the overall rigidity of the toxin by forming both interdomain and intradomain connections, referred to by the roman numerals I–V. The first domain is stabilized by two disulfide bonds formed by Cys18–Cys34 (I) and Cys22–Cys30 (II), both connecting α1 and α2. In the second domain, a disulfide bond, Cys61–Cys71 (IV), connects α3 and α4 and a disulfide bond formed by Cys57–Cys89 (V) tethers the C-terminus to α3. An interdomain disulfide bond formed by Cys10–Cys51 (III) connects 3_10_-N to α3 and contributes to the overall rigidity of Mu8.1 (Figs. 1 ▸ *a* and 1 ▸ *b*).

### 3.2. Lys55 at the Mu8.1 dimer interface has a low predicted p*K*_a_ value

**Figure 2:**
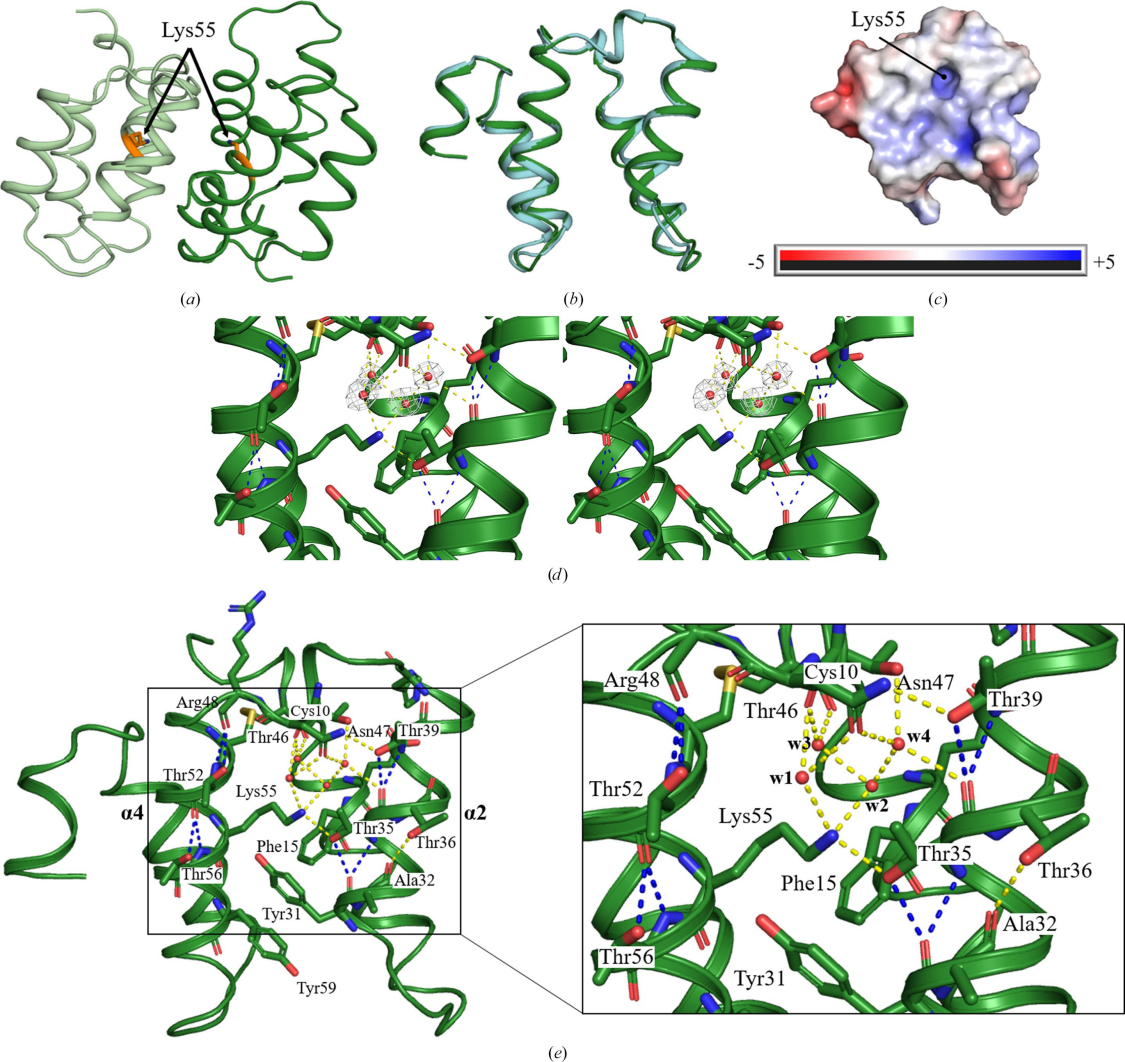
Structural analysis of the high-resolution crystal structure of Mu8.1. (*a*) Mu8.1 (dark green) forms the previously identified homodimeric conformation with a symmetry-related molecule (pale green). Lys55 is shown in orange. (*b*) Superimposition of Mu8.1 (PDB entry 8amy, dark green cartoon) with PDB entry 7px2 (cyan cartoon; Hackney *et al.*, 2023 ▸). (*c*) Surface electrostatics (red, negative; blue, positive) of Mu8.1 shown in the same orientation as in (*b*), (*d*) and (*e*). (*d*) A stereoview of the electron density for the four water molecules (red spheres) coordinated around Lys55. The electron-density map is contoured at the 1σ level. (*e*) The water network (yellow dashed lines) around Lys55 and the threonine residues involved in bifurcated hydrogen bonds (blue dashed lines). The side-chain OH groups and backbone NH groups of Thr35, Thr36, Thr39, Thr52 and Thr56 act as hydrogen-bond donors, while the backbone carbonyl groups of Tyr31, Ala32, Thr35, Arg48 and Thr52 act as acceptors. Water molecules are presented as red spheres and labeled w1–w4. N, O and S atoms are colored blue, red and yellow, respectively.

The determined structure revealed a homodimer of Mu8.1 between crystallographic symmetry-related molecules, forming a dimer interface identical to that which we previously reported in Hackney *et al.* (2023 ▸) (Fig. 2 ▸ *a*). The conserved Lys55 is located at the dimer interface and is thus shielded from solvent (Fig. 2 ▸ *a*; see further discussion below). The previously published structures (PDB entries 7px1 and 7px2; Hackney *et al.*, 2023 ▸) accommodate one and three homodimers in the asymmetric unit, respectively. All observed homodimers form the same protomer-to-protomer orientation. Superimposition of the protomer from PDB entry 7px2 with the Mu8.1 structure reported in this work results in an r.m.s.d. of 0.69 Å for C^α^ atoms covering 80 residues (Fig. 2 ▸ *b*). Due to our consistent observation of Mu8.1 in the same homodimeric conformation in three independent crystal conditions, the dimer conformation is likely to be of biological relevance.

The p*K*_a_ value of ionizable amino-acid residue side chains depends on the surrounding microenvironment. Ionizable side chains in a polar microenvironment of the protein will tend to have the same p*K*_a_ as in water, while in a hydrophobic microenvironment these side-chain p*K*_a_ values will tend to shift towards the neutral state (Isom *et al.*, 2011 ▸). The Mu8.1 dimer interface is largely hydrophobic, with a pocket accommodating the strictly conserved Lys55 (Fig. 2 ▸ *c*; Hackney *et al.*, 2023 ▸). This feature of the structure prompted us to predict the p*K*_a_ values of all charged amino-acid residues of Mu8.1 (summarized in Table S1) using the *PROPKA* algorithm as described in Section 2. Notably, the predicted side-chain p*K*_a_ value of Lys55 (8.16) stands out as that which diverges most from the side-chain p*K*_a_ of the free amino acid (10.5). The relatively low side-chain p*K*_a_ could imply that Lys55 is uncharged in the dimer, thus rationalizing how it can be accommodated at the hydrophobic dimer interface.

### 3.3. Bifurcated hydrogen bonds may contribute to the lowered p*K*_a_ value of Lys55

To determine the molecular origin of the predicted low side-chain p*K*_a_ for Lys55, we inspected the structural details of the dimer interface. The high-resolution structure of Mu8.1 obtained in this work allowed us to identify a tightly coordinated hydrogen-bond network around Lys55. Several new water molecules were discovered in this higher resolution structure compared with the previous structures PDB entries 7px1 and 7px2 (Supplementary Fig. S1). Fig. 2 ▸(*d*) shows the electron-density map for the four water molecules refined in the vicinity of Lys55 in the high-resolution structure. Here, Lys55 shows fully saturated hydrogen-bond formation with two water molecules (w1 and w2) and the side-chain hydroxyl group of Thr35 (Fig. 2 ▸ *e*). The water networks extends from w2 to waters w3 and w4. Water w3 is also coordinated by the carbonyl groups in the backbones of Cys10 and Thr46. Water w4 forms hydrogen bonds to the hydroxy group of Thr46, the side-chain amide of Asn47 and the backbone carbonyl of Thr35. The latter residue forms a bifurcated hydrogen bond with the backbone carbonyl group of Tyr31. Four additional threonine residues (Thr36, Thr39, Thr52 and Thr56) are found within the otherwise nonpolar dimer interface. All five threonine residues form bifurcated hydrogen bonds from their side-chain OH group to the backbone carbonyl group of the residue at position *i* + 4 relative to the threonine. Thus, these five threonine residues act as donors, while the residues found at the *i* + 4 helical position, Tyr31, Ala32, Thr35, Arg48 and Thr52, act as acceptors (Fig. 2 ▸ *e*). All five threonine residues are trapped in the +*gauche* conformation with an χ_1_ angle close to −60°, as expected for this type of interaction (Feldblum & Arkin, 2014 ▸). This type of bifurcated hydrogen bonding is particularly strong (Feldblum & Arkin, 2014 ▸) and has previously been suggested to allow threonine and serine residues to reside in transmembrane α-helices (Engelman & Steitz, 1981 ▸). Moreover, we noticed π–π stacking of the aromatic rings of Tyr31 and Phe15 (Fig. 2 ▸ *e*). Overall, we propose that the combination of this side-chain stacking and the observed hydrogen-bonding network creates a hydrophobic microenvironment surrounding Lys55 that results in a lowering of the p*K*_a_ of its ɛ-amino group.

### 3.4. A Zn^2+^ ion-binding site mediates crystal contacts between two Mu8.1 dimers

**Figure 3:**
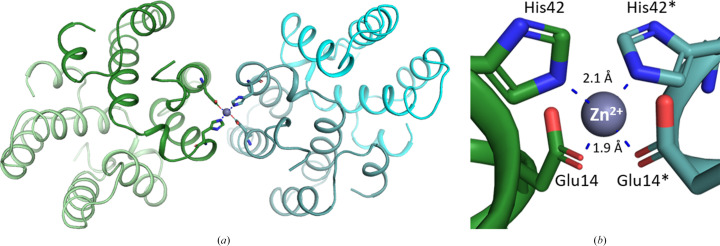
(*a*) Cartoon representation of the protomers involved in Zn^2+^ (gray sphere) binding (dark green and teal cartoons), which are distinct from the promoters comprising the previously described dimer (light green/dark green cartoons and teal/cyan cartoons). (*b*) Residues involved in the coordination of a Zn^2+^ ion (gray sphere) between two Mu8.1 protomers are shown as dark green and teal sticks. Intermolecular distances are shown as blue dashed lines and are labeled in Å.

The present high-resolution crystal structure of Mu8.1 also revealed a possible zinc (Zn^2+^) ion-binding site (Fig. 3 ▸ *a*). The putative Zn^2+^ ion is located on the surface of the first domain (α1 and α2) and mediates a crystal contact between equivalent sites in a symmetry-related molecule that differs from the previously described dimer interface (Fig. 3 ▸ *a*). The divalent cation ion is coordinated by His42 and Glu14 in a classical tetrahedral geometry, with coordination distances corresponding to a Zn^2+^ site (Fig. 3 ▸ *b*; Alberts *et al.*, 1998 ▸). The previously determined structure of Mu8.1 (PDB entry 7px1; Hackney *et al.*, 2023 ▸) included divalent binding sites for Cd^2+^ ions, which were introduced from the crystallization conditions and were located at equivalent positions to the metal site presented here (Hackney *et al.*, 2023 ▸). However, no additional divalent ions were added in the present crystallization conditions, and hence the observed metal ion must have been carried along in the purification process: either a cytoplasmic Zn^2+^ ion or an Ni^2+^ ion originating from the Ni–NTA purification step. The complex coordination and the metal–ligand distances are equivalent to the typical tetrahedral coordination found for a Zn^2+^ site in biological molecules (Dudev & Lim, 2000 ▸; Kuppuraj *et al.*, 2009 ▸). It is therefore likely that a Zn^2+^ ion chelates to the surface of Mu8.1 under physiological conditions. In connection to this, we note that previous dose–response experiments performed on HEK293 cells transiently overexpressing Cav2.3 revealed that trace metals, including Zn^2+^ and Cu^2+^, modulate the voltage-dependent gating of Cav2.3, with reported IC_50_ values of 1.3 µ*M* and 18.2 n*M*, respectively (Shcheglovitov *et al.*, 2012 ▸). The ability of Mu8.1 to coordinate Zn^2+^ may therefore comprise an additional level of regulation in terms of Cav2.3 inhibition. Still, a potential physiological function for Zn^2+^ binding by Mu8.1 remains speculative.

### 3.5. Implications of the Mu8.1 structure for target interactions

The current findings have implications for Mu8.1 target interactions. The consistent observation of Mu8.1 in identical homodimeric crystal conformations suggests that this dimer is of biological relevance. In connection to this, we propose that the bifurcated hydrogen bonds formed by threonine residues at the dimer interface and the water network formed around Lys55 contribute to the hydrophobicity observed at the dimer interface. Analytical gel-filtration and SAXS data also show that Mu8.1 exists as a dimer in solution at concentrations above 1 µ*M* (Hackney *et al.*, 2023 ▸). With interaction studies between Mu8.1 and Cav2.3 conducted at concentrations of 1– 10 µ*M* (Hackney *et al.*, 2023 ▸), binding to this target is likely to take place in the dimeric state.

Still, it cannot be ruled out that Mu8.1 may bind targets (for example if other physiological targets exist in addition to Cav2.3) in a monomeric state. A potential indication of this binding mode comes from an analysis of the interaction between con-ikot-ikot and AMPAR. As described in Hackney *et al.* (2023 ▸), Mu8.1 shows high structural similarity to con-ikot-ikot. Despite the lack of AMPAR binding by Mu8.1 (Hackney *et al.*, 2023 ▸), we hypothesized that the structural resemblance to con-ikot-ikot may predict the region of Mu8.1 that is involved in target binding. Thus, we superimposed Mu8.1 with con-ikot-ikot as observed in complex with AMPAR (PDB entry 4u5b; Chen *et al.*, 2014 ▸; Supplementary Fig. S2). This analysis revealed that the hydrophobic interface of Mu8.1, which is shielded in the dimer, is equivalent to the face of con-ikot-ikot involved in AMPAR binding. Provided that the dimer interface is involved in target binding in a monomeric state, a functional role of Lys55 can be envisaged in which a conformational change in the protein would result in the side chain acquiring a full positive charge for electrostatic interaction with the target. This scenario would not be unusual, given that charged residues located in a pocket or in the hydrophobic interior of a protein, such as Lys55, often serve a functional role in protein–protein interactions (or as catalytic residues because of increased reactivity; Isom *et al.*, 2011 ▸; Hacker *et al.*, 2017 ▸). Moreover, conversion from an inactive dimer to an active monomer formed through mechanisms involving, for example, proteolytic cleavage, dilution or membrane inter­action has been observed for other toxins, such as the aerolysin toxin from *Aeromonas hydrophila* (Fivaz *et al.*, 1999 ▸) and phospholipase A_2_ from *Bothrops jararacussu* venom (Ruller *et al.*, 2003 ▸). If a dimer–monomer equilibrium indeed regulates Mu8.1 target binding, monomerization could for instance be induced by specific physiological conditions in the prey or by a concentration-dependent mechanism (*i.e.* dilution).

Overall, the present structure provides detailed insight into several features of the Mu8.1 dimer interface, with implications for target interactions. Future investigations will be aimed at discerning the exact binding mode of Mu8.1, for example through co-crystallization or docking studies.

## Supplementary Material

PDB reference: Mu8.1, 8amy

Supplemetary Table and Figures. DOI: 10.1107/S2053230X23007070/ow5036sup1.pdf

## Figures and Tables

**Table 1:** Crystallization information

Method	Hanging-drop vapor diffusion
Plate type	24-well XRL plate (Molecular Dimensions)
Temperature (K)	294
Protein concentration (mg ml^−1^)	5
Buffer composition of protein solution	200 m*M* NH_4_HCO_3_ buffer pH 7.8
Composition of reservoir solution	18%(*w*/*v*) PEG 5000 MME, 0.2 *M* ammonium sulfate, 0.1 *M* MES pH 6.5
Volume and ratio of drop	1:1, 2 µl total
Volume of reservoir (µl)	500

**Table 2:** Data collection and processing Values in parentheses are for the outer shell.

Diffraction source	BioMax, MAX IV
Wavelength (Å)	0.9763
Temperature (K)	100
Detector	EIGER 16M†
Crystal-to-detector distance (mm)	285.99
Rotation range per image (°)	0.1
Total rotation range (°)	360
Exposure time per image (s)	0.011
Space group	*I*4_1_22
*a*, *b*, *c* (Å)	52.86, 52.86, 137.43
α, β, γ (°)	90, 90, 90
Mosaicity (°)	0.061
Resolution range (Å)	34.62–1.67 (1.73–1.67)
Total No. of reflections	95474 (3637)
No. of unique reflections	11643 (1052)
Completeness (%)	98.87 (91.62)
Multiplicity	8.2 (3.5)
〈*I*/σ(*I*)〉	16.59 (1.03)‡
*R*_meas_	0.071 (>1)
*R*_p.i.m._	0.023 (0.62)
CC_1/2_	0.999 (0.345)
Overall *B* factor from Wilson plot (Å^2^)	30.22§

† Casanas *et al.* (2016 ▸).

‡ *I*/σ(*I*) falls below 2.0 at 1.85 Å; we used CC_1/2_ as an indicator of the resolution.

§ No anomalies were detected.

**Table 3:** Structure solution and refinement Values in parentheses are for the outer shell.

Resolution range (Å)	34.62–1.67 (1.73–1.67)
Completeness (%)	98.87 (91.62)
No. of reflections, working set	11639 (1050)
No. of reflections, test set	582 (70)
Final *R*_work_	0.169 (0.316)
Final *R*_free_	0.200 (0.302)
No. of non-H atoms	
Protein	708
Ligand	53
Water	70
R.m.s. deviations	
Bond lengths (Å)	0.015
Angles (°)	1.67
Average *B* factors (Å^2^)	
Protein	40.0
Ligands	50.6
Solvent	46.4
Ramachandran plot	
Most favored (%)	96.4
Allowed (%)	3.6
Outliers (%)	0.00
