# Supplementary material for: High-resolution crystal structure of the Mu8.1 conotoxin from Conus mucronatus
Source: Acta Crystallogr F Struct Biol Commun. 2023 Aug 29;79(Pt 9):240–6. doi: 10.1107/S2053230X23007070 (PMC10478764; doi:10.1107/S2053230X23007070)
Supplement: Supplementary file 1 [file f-79-00240-sup1.pdf]

STRUCTURAL BIOLOGY COMMUNICATIONS

Volume 79 (2023)

Supporting information for article:

High-resolution crystal structure of the Mu8.1 conotoxin from *Conus mucronatus*

Emilie Müller, Celeste Menuet Hackney, Lars Ellgaard and Jens Preben Morth

Table S1 Predicted pK_a values of amino acid side chains in Mu8.1

Amino acid	Residue	pK_a	Model-pK_a
ASP	28	3.38	3.8
ASP	45	2.95	3.8
ASP	50	2.71	3.8
ASP	69	2.61	3.8
ASP	82	3.56	3.8
ASP	85	3.25	3.8
GLU	14	3.5	4.5
GLU	21	4.59	4.5
GLU	68	3.33	4.5
GLU	87	4.56	4.5
HIS	9	6.98	6.5
HIS	29	6.58	6.5
HIS	42	5.45	6.5
HIS	70	6.31	6.5
LYS	55	8.16	10.5
LYS	66	10.48	10.5
LYS	73	11.37	10.5
ARG	11	11.99	12.5
ARG	16	13.98	12.5
ARG	33	12.32	12.5
ARG	48	13.35	12.5
ARG	60	12.22	12.5

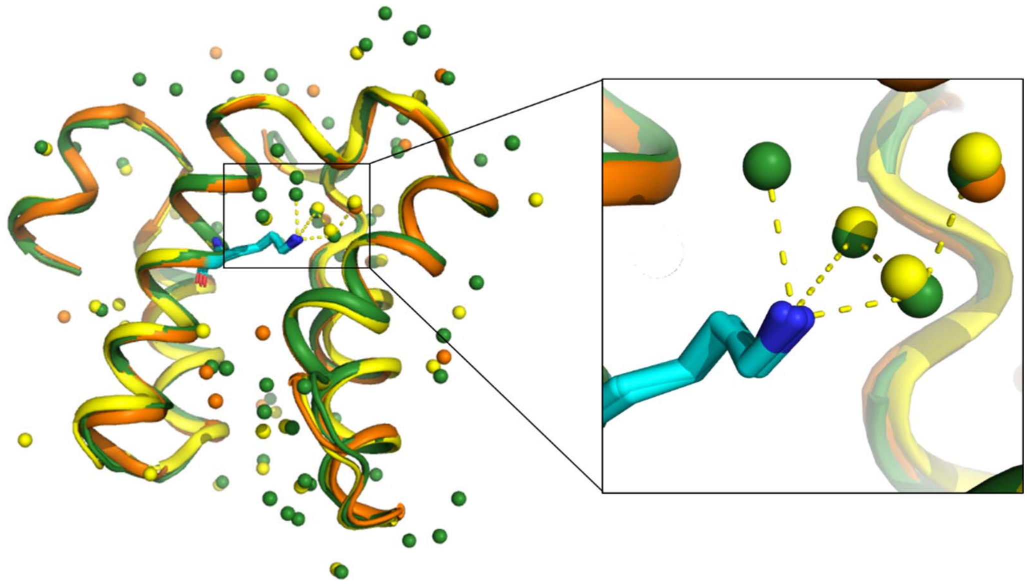

Figure S1 Alignment of 8AMY (green), 7PX1 (orange) and 7PX2 (yellow) with refined water molecules shown in spheres. Lysine 55 (Lys55) is shown in cyan sticks with nitrogen highlighted in dark blue. The four water molecules coordinated around Lys55 are visualized on the right zoom image.

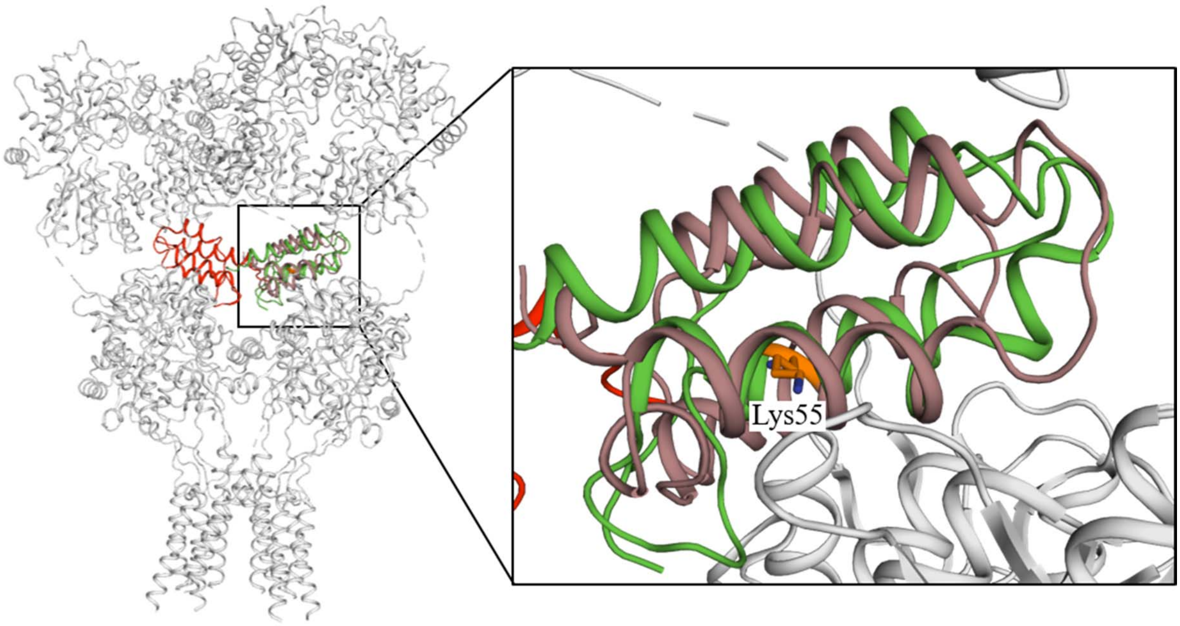

Figure S2 Superimposition of Mu8.1 with con-ikot-ikot in the GluA2 AMPA receptor complex. The AMPA receptor (PDB code 4U5B; Chen et al., 2014) is presented in grey cartoon, and con-ikot-ikot subunits are presented as red and brown cartoons. Mu8.1 is presented in green cartoon with Lys55 highlighted in orange sticks.
